# Serious illness conversations and quality of end-of-life care in patients with hematological malignancies—a retrospective quality improvement study

**DOI:** 10.1007/s00520-025-09855-2

**Published:** 2025-08-27

**Authors:** Cæcilie Borregaard Myrhøj, Rachelle Bernacki, Selma Bjerre-Bertelsen, Juliet Jacobsen, Jenny Klintman, Mary Jarden, Christoffer Johansen, Annika von Heymann, Stine Novrup Clemmensen

**Affiliations:** 1 Department of Hematology, Copenhagen University Hospital, Rigshospitalet, Copenhagen Denmark; 2 CASTLE - Cancer Survivorship and Treatment Late Effects Research Unit, Department of Oncology, Copenhagen University Hospital, Rigshospitalet, Copenhagen Denmark; 3 Department of Clinical Medicine, University of Copenhagen, Copenhagen, Denmark; 4 Department of Clinical Sciences Lund, Medical Oncology, Lund University, Lund, Sweden; 5 Harvard Medical School, Boston, USA; 6 Massachusetts General Hospital, Boston, USA; 7 Department of Supportive Oncology, Dana Farber Cancer Institute, Boston, USA; 8 Palliative Care and Geriatric Medicine, Mass General Brigham, Boston, USA; 9 Department of Internal Medicine, Herlev and Gentofte Hospital, Copenhagen, Denmark; 10 Palliative Care Research Unit, Bispebjerg and Frederiksberg Hospital, Copenhagen, Denmark; 11 The Institute for Palliative Care, Lund, Sweden

**Keywords:** Palliative care, Terminal care, Hematology, Retrospective studies, Communication, Advance care planning

## Abstract

**Purpose:**

Patients with hematological malignancies frequently receive aggressive, poor-quality end-of-life care. In oncology, serious illness conversations conducted early in the illness trajectory, focusing on patients’ goals, values, and priorities, have been associated with improved end-of-life care and decreased symptoms of anxiety and depression. Yet, evidence of such supportive interventions’ impact in hematology remains limited. This study explores the association between receiving a serious illness conversation and the quality of end-of-life care and the timing of serious illness conversations in patients with hematological malignancies.

**Methods:**

Single-center retrospective quality improvement study. Data on receipt of serious illness conversations and end-of-life care (hospitalizations, specialized palliative care referrals, place of death, receipt of anticancer treatment) were extracted from electronic healthcare records. Logistic regression, adjusted for sex, age, and diagnosis examined the differences between patients who did and did not receive a serious illness conversation. The study included patients with hematological malignancies, who died between 2020 and 2022 and received anticancer treatment within the last 12 months at a university hospital in Denmark.

**Results:**

Among 311 patients (median age 74 years, 43% female), 63 (20%) received a serious illness conversation. Patients receiving conversations had significantly higher odds of referral to specialized palliative care (OR 2.67, 95% CI [1.44; 4.91]) and lower odds of receiving anticancer treatment within 30 days (OR 0.19, 95% CI [0.10; 0.37]) and 14 days (OR 0.21, 95% CI [0.09; 0.46) before death.

**Conclusion:**

Serious illness conversations are associated with reduced aggressive end-of-life anticancer treatment and increased referrals to specialized palliative care.

## Introduction

Despite significant advances in the treatment of hematological malignancies, most remain associated with high mortality, necessitating a strong focus toward end-of-life care [1, 2]. Compared to patients with solid tumors, patients with hematological malignancies receive more aggressive treatments and low-quality end-of-life care, including more frequent emergency room visits, end-of-life hospitalizations, anticancer treatments, and less often specialized palliative care services [3–7]. Complicating decision-making, many patients with hematological malignancies overestimate their chances of cure [8–12], leading to lower hospice utilization and increased hospitalizations [13]. A crucial step in avoiding futile treatments and reducing suffering may be effective communication between patients, their caregivers, and healthcare providers about prognosis, values, and goals of care.

The Serious Illness Care Program [14] was developed to enhance quality of communication through structured conversation tools, clinician training, and system changes. In a cluster-randomized trial involving 286 cancer patients, the program resulted in more and earlier conversations centered on values, goals, prognostic understanding, and in reductions in symptoms of anxiety and depression [15, 16]. Another recent randomized trial, including 25% with lymphoma and multiple myeloma, in the USA demonstrated that increasing the number of serious illness conversations reduced use of out-patient systemic therapy at end of life [17]. The few retrospective studies that have examined the association of such conversations with quality of end-of-life care, specifically in patients with hematological malignancies, found significantly improved documentation of goals-of-care [18], lower odds of intensive care unit admission, and earlier hospice enrolment [19]. However, these studies did not explore an association with anticancer treatment, referral to specialized palliative care, or place of death.

### Aim

We utilized data from the Danish adaptation and implementation of the Serious Illness Care Program in a hematological outpatient setting [20, 21] to explore the association between receiving a serious illness conversation and quality of end-of-life care, and the timing of serious illness conversations in patients with hematological malignancies.

## Methods

The reporting of this study adheres to the Revised Standards for Quality Improvement Reporting Excellence (SQUIRE 2.0) [22].

### Study design and population

We conducted a single-center retrospective quality improvement study at the Department of Hematology, Copenhagen University Hospital, Rigshospitalet. Patients seen by physicians who had received Serious Illness Conversation training (see intervention description below) were screened. This study included all patients ≥ 18 years of age who died from a hematological malignancy between January 2020 and December 2022 and had received anticancer treatment at the department within 12 months before death. Patients were excluded if they had been formally discharged from our hospital’s care and transferred to another hospital before death, received only low-intensity palliative anticancer treatments (e.g., hydroxyurea or mercaptopurine), or underwent allogeneic hematopoietic stem cell transplantation as their final treatment, as transplantation clinicians were not part of the implementation.

### Intervention and implementation

In 2020, a structure for serious illness conversations was implemented as the new standard of practice, including diagnosis-specific guidelines for conversation timing, allocated time for conversations, and standardized mandatory documentation templates in medical records. The diagnosis-specific guidelines recommended offering serious illness conversations (1) to patients with multiple myeloma, high-risk myelodysplastic syndrome, acute leukemia, and aggressive lymphoma (e.g., T cell lymphoma and CNS lymphoma) at the time of diagnosis; (2) to patients with myelofibrosis when weekly transfusions were required, and (3) to all patients at relapse and transition to palliative care. To train all clinicians, five separate 8-h interprofessional Serious Illness Care training days were conducted for the department’s physicians and nurses. Each clinician attended one full-day session, which included simulation-based skills practice with professional actors and was guided by a structured Serious Illness Conversation Guide [21, 23]. Following the training, clinicians initiated structured conversations with patients and caregivers, addressing prognosis, concerns, hopes, treatment goals, and care preferences. Interprofessional group supervision led by a senior palliative care physician and the hospital chaplain supported conversation, ethical dilemmas, and emotional support. Additional support included preparatory materials for patients and caregivers.

### Data collection

In 2023, after 3 years of implementation (2020–2022), records of potentially eligible patients were identified through electronic medical records and systematically assessed for eligibility by authors CBM, SNC, and SBB. In 2024, data were extracted from eligible patients’ records, including serious illness conversation documentation and end-of-life care outcomes. Dates of serious illness conversation documentation were recorded, and the number of days between conversations and death was calculated. Hospital days within the last 30 days of life were recorded, as well as the date of the last intravenous, intrathecal, subcutaneous, or oral administration of anticancer treatment, number of treatment lines given since diagnosis, documentation of do-not-resuscitate, referral to specialized palliative care (including referral to hospices and palliative care units), and place of death (hospital, intensive care unit, home (including nursing homes), specialized palliative care facilities, municipal facility e.g., rehabilitation center, or “unknown” if not documented).

Sex, age, and hematological diagnosis were extracted, and diagnoses were categorized into “multiple myeloma/amyloidosis,” “lymphoma,” and “acute leukemia/myelodysplastic syndrome/myeloproliferative neoplasms.” These covariates have previously been shown to influence end-of-life outcomes [24–27].

### Statistical analysis

Frequencies and percentages were used to describe characteristics and end-of-life care outcomes for patients who received a serious illness conversation and those who did not (Table 1). To explore associations between receiving a serious illness conversation and end-of-life care outcomes, we ran logistic regression models adjusted for potential confounding covariates (age, sex, and diagnosis), estimating odds ratios and 95% confidence intervals (CI) for the end-of-life outcomes: anticancer treatment, hospitalization, referral to specialized palliative care, do-not-resuscitate and place of death (Table 2). Analyses were conducted in R [28].

**Table 1:** Patient characteristics and end-of-life care outcomes

**Characteristics**	**All participants** (*N* = 311)	**No serious illness conversation (SIC)** (*N* = 248)	**Received serious illness conversation (SIC)** (*N* = 63)
**Age in years, median (IQR, min–max)**	74 (12, 25–94)	74 (12, 25–91)	76 (13, 25–94)
**Lines of treatment, median (IQR, min–max)**	2 (2, 1–12)	1 (2, 1–10)	3 (3, 1–12)
**Sex (female),* N* (%)**	134 (43)	104 (42)	30 (48)
**Diagnosis, *N* (%)**			
Multiple myeloma/amyloidosis	107 (34)	66 (27)	41 (65)
Lymphoma	111 (36)	104 (42)	7 (11)
Acute leukemia/MDS/MPN	93 (30)	78 (31)	15 (24)
**Place of death**, ***N* (%)**			
Specialized palliative care	72 (23)	51 (20)	21 (33)
Hospital	129 (41)	107 (43)	22 (35)
Intensive care unit	28 (9)	26 (10)	2 (3)
Home	56 (18)	41 (16)	15 (24)
Municipality	12 (4)	10 (4)	2 (3)
Unknown	14 (5)	13 (5)	1 (2)
**Hospitalization within the last 30 days of life, *N* (%)**	260 (84)	214 (86)	46 (73)
Days in hospital: 1 ≤ 14 days	137 (53)*	104 (49)	33 (72)
Days in hospital: 15–30 days	123 (47)*	110 (51)	13 (28)
**Anticancer treatment 0 ≤ 30 days before death, *N* (%)**^†^	167 (54)	151 (61)	16(25)
Hereof 1 ≤ 14 days before death	101 (60)^‡^	92 (61)	9 (56)
**Referral to specialized palliative care, *N* (%)**	139 (45)	99 (40)	40 (63)
**Do-not-resuscitate documented**, ***N* (%)**	269 (87)	212 (86)	57 (90)
**Days to event, median (IQR, min–max)**			
Days from last anticancer treatment to death*	28 (53, 0–362)	23 (43, 0–362)	54 (98, 0–310)
Days from “Do-not-resuscitate” to death	26 (57, 0–886)	23 (49, 0–868)	54 (112, 1–886)
Days from referred to specialized palliative care to death	18 (44, 1–393)	15 (28, 1–277)	35 (107, 2–393)
Days between SIC 1 and death			204 (296, 2–720)
Days between SIC 2 and death			129 (113, 12–229)

*MDS* = Myelodysplastic syndrome; *MPN* = Myeloproliferative neoplasms

*Percentage calculated based on the number of patients hospitalized within the last 30 days of life

^†^Palliative anticancer treatment (Hydroxyurea/Thioguanine) excluded

^‡^Percentage calculated based on the number of patients who received anticancer treatment ≤30 days before death

## Results

**Fig. 1:**
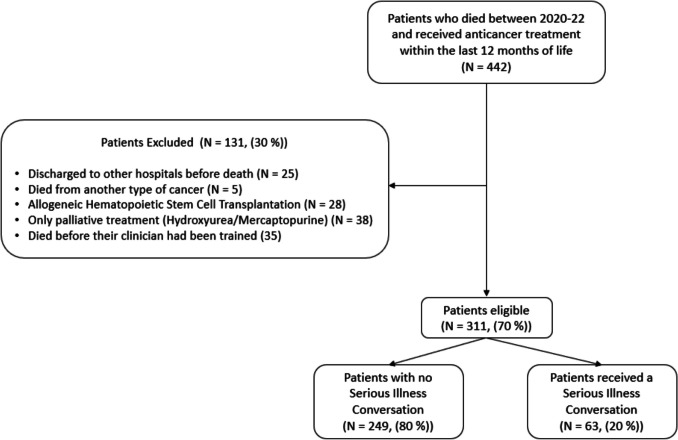
Flowchart of inclusion

A total of 311 patients were included (Fig. 1), 43% were female, with a median age of 74 years. Thirty-four percent had multiple myeloma or amyloidosis, 36% lymphoma, 30% acute leukemia, myelodysplastic syndrome, or myeloproliferative neoplasms. Patients had received a median of two lines of treatment since diagnosis (Table 1). Among all patients, 54% received anticancer treatment within the last 30 days of life, 84% were hospitalized within the last 30 days, and 45% were referred to specialized palliative care. Sixty-three patients (20%) had documented receipt of at least one serious illness conversation, with a median of 204 days before death. Twenty-two patients received a second conversation, with a median of 129 days before death (Table 1).

Of the 298 patients with known place of death, the majority died in hospital (*n* = 129), followed by specialized palliative care facilities (including hospice) (*n* = 72), home (*n* = 56), intensive care units (*n* = 28), and municipal facilities (*n* = 12) (Table 1).

Receiving a serious illness conversation was associated with significantly lower receipt of anticancer treatment in the last 30 days (OR 0.19, 95% CI [0.10; 0.37]) and 14 days of life (OR 0.21, 95% CI [0.09; 0.46) and significantly more referrals to specialized palliative care (OR 2.67, 95% CI [1.44; 4.91]) (Table 2). The associations between receiving a serious illness conversation and hospitalization within the last 30 days of life and documentation of do-not-resuscitate status or place of death did not reach statistical significance (Table 2).

**Table 2:** Associations between serious illness conversations and end-of-life care outcomes

End-of-life care outcome	OR	95% CI	*p* value
**Anticancer treatment**			
0 ≤ 30 days before death	0.19	0.10; 0.37	** < 0.001**
1 ≤ 14 days before death	0.21	0.09; 0.46	** < 0.001**
**Hospitalization**			
Hospitalized within the last 30 days of life	0.51	0.25; 1.04	0.062
**Referral to specialized palliative care**	2.66	1.44; 4.91	**0.002**
**Do-not-resuscitate documented**	1.57	0.59; 4.14	0.362
**Place of death**			
Hospital	Reference	Reference	Reference
Specialized palliative care	2.03	0.97; 4.26	0.062
Intensive care unit	0.42	0.09; 2.03	0.280
Home	1.71	0.76; 3.82	0.191
Municipality	1.28	0.22; 7.35	0.784

Note: Patients not receiving serious illness conversations are the reference group for all analyses. Odds ratios (OR) are adjusted for age, sex, and diagnosis

Bold values indicate statistically significant associations (*p* < 0.05)

## Discussion

In this retrospective cohort study of serious illness conversations in patients with hematological malignancies, we found that 20% of patients received at least one conversation a median of 6.7 months prior to death. Receiving a conversation was associated with significantly lower odds of anticancer treatment in the last 30 and 14 days of life, as well as significantly higher odds of referral to specialized palliative care. Additionally, there was a trend toward more patients who had received a conversation dying at home, in specialized palliative care facilities, or municipal facilities, and fewer in intensive care units.

The high proportion of patients receiving anticancer treatment (54%) in the last 30 days of life and the association of receiving serious illness conversations with less end-of-life anticancer treatment, in our study highlight an important opportunity to reduce treatment intensity at end of life. A previous prospective study in the USA of 160 patients with acute leukemia reported that 65.9% of the 44 patients who died during follow-up received anticancer treatment in the last 30 days of life [29]. Similar to our study, patients who participated in timely conversations about illness understanding and treatment goals had lower odds of receiving anticancer treatment at end of life [29]. A retrospective study of 319 deceased patients with various hematological malignancies found that conversations about goals of treatment were an independent predictor of end-of-life outcomes and positively impacted anticancer treatment [30]. Other retrospective studies of various hematological malignancies reported lower rates (16–45%) of anticancer treatment at end of life, independent of a serious illness conversation based on data from 2009 to 2016 in the USA (*n* = 113 [31]), Canada (*n* = 319 [30]), and France (*n* = 46,629 [24]). Advancements in therapies since that period may explain the higher prevalence of end-of-life treatment in our study. Yet, our study was conducted during the COVID-19 pandemic. Patients with hematological malignancies and COVID-19 experienced higher mortality [32, 33], and acute death due to COVID-19 infections may have led to more anticancer treatment close to death. In fact, the pandemic highlighted an increased need for structured approaches, such as serious illness conversations, due to the heightened mortality.

In our study, patients who received a conversation had received more lines of treatment since diagnosis, likely because more patients with multiple myeloma, a malignancy often associated with more available treatment lines than acute leukemia and lymphomas, received conversations. We did not extract data on date of diagnosis and therefore cannot compare survival between groups. Our findings could, however, suggest that conversations were initially offered to patients with longer treatment trajectories, aligning with the natural progression of an implementation process where early adopters may prioritize patients who are more obviously suitable before extending conversations to earlier stages of disease. However, a median of three treatment lines (IQR 1–12) suggests that conversations were often initiated early.

We found a median of 204 days between the first serious illness conversation and death, reflecting a successful shift to earlier conversation timing. Receiving a serious illness conversation was significantly associated with higher odds of referral to specialized palliative care, with a median of 35 days from referral to death compared to 15 days for those without a conversation. This earlier conversation timing and referral may provide patients with more time in specialized palliative care, potentially enhancing the quality of end-of-life care. The role of timely discussions in optimizing end-of-life care has previously been documented in a retrospective study of 383 patients, where goals-of-care conversations conducted in outpatient settings with hematologists more than 30 days before death, compared to those held within 30 days, were associated with fewer intensive care unit admissions and longer enrolment in hospice care [19]). However, it is noteworthy that we found conversations conducted at a median time of approximately 6 months before death associated with changed end-of-life care. Nevertheless, another retrospective analysis of 332 deceased patients from a prospective multisite study found end-of-life discussions within the last 4 months of life associated with prolonged hospice stays [34]. In our study, the trend toward more home deaths may be driven by increased specialized palliative care referrals, as 60% of patients who died at home and had serious illness conversations had been referred to specialized palliative care compared to 41% of those without a conversation. Mixed outcomes across studies on other end-of-life outcomes suggest the need for further exploration of the timing and frequency of these conversations to gain insights into optimizing end-of-life care for patients with hematological malignancies.

Barriers among hematological clinicians to early integration of specialized palliative, such as fear of taking away hope, prognostic uncertainty, and misconceptions about palliative care as only end-of-life care, have historically led to underuse of specialized palliative care services [35–37]. Although all clinicians received serious illness communication training, a significant proportion of patients did not receive a serious illness conversation before death. This underscores the complex influence of clinical culture on adopting serious illness conversation practices during implementation and the intensive treatment culture within hematology. A French cross-sectional national study found that while most hematologists recognize the importance of end-of-life discussions, only a minority routinely address these topics, reflecting a knowledge-practice gap [38]. A qualitative study with 30 clinicians and leaders from five healthcare systems implementing the Serious Illness Care Program in the United States revealed that clinicians often view these conversations as solely concerning end-of-life planning, contributing to hesitancy [39]. Another qualitative study involving 14 physicians from cardiology, endocrinology, hematology, and palliative care, at two Swedish hospitals implementing the Serious Illness Care Program, highlighted concerns about timing, emotional harm, and undermining hope, which may delay these conversations [40]. In our study, most patients who received a serious illness conversation were diagnosed with multiple myeloma [65%] and were therefore treated by the same team of clinicians. Some of the clinicians treating multiple myeloma were involved in the process of adapting the Serious Illness Care Program to a Danish cultural setting, including participation in workshops with patients and caregivers, and were the first to receive training, which may partly explain the higher implementation of conversations in this group. Moreover, multiple myeloma is incurable from the time of diagnosis; thus, the treatment approach might generally be more palliative compared to that of acute leukemia or aggressive lymphoma, which may have further facilitated the integration of serious illness conversations into clinical practice. This suggests that we may have identified a subgroup of clinicians who were more engaged in conducting these conversations and might have different practices around end-of-life care. Yet, even within the multiple myeloma group, where uptake of conversations was highest, not all patients received a conversation before death, reflecting variability in implementation. This variation may be influenced by differences in clinician engagement, integration of the training into practice, or patient-level factors such as comorbidities or disease trajectory, although our data do not allow further exploration of these potential explanations. Shifting the focus from “end-of-life planning” to “understanding what matters most to patients” may help bridge the gap between knowledge and practice [39]. Further research is needed on the use of serious illness conversations in diagnoses like acute leukemia, where prognostic uncertainty is high, and the potential impact may be substantial.

The small percentage of patients receiving serious illness conversations may also be due to patients declining the invitation. A multi-site cross-sectional study from Canada found that patients may decline due to concerns about misaligned values, emotional distress, not feeling mentally ready, or insufficient time to address complex issues [41]. A qualitative study with 44 patients, caregivers, and clinicians in a randomized trial identified barriers, including patients’ coping strategies, such as focusing on the present and maintaining optimism, and clinicians’ hesitancy and discomfort with discussing prognostic information, which discouraged patient engagement [42]. Other studies have highlighted racial and ethnic disparities in the willingness to engage in serious illness conversations or advance care planning discussions [43–46], as well as prevalent symptoms of anxiety and depression in the patients, which may affect their willingness to participate [47, 48].

### Strengths and limitations

Our study has several strengths and limitations. First, the retrospective nature of the data limits our ability to establish causality. While associations have been identified, we cannot determine whether observed differences are due to the conversations or other unmeasured factors. Our study was also limited by the lack of access to data on racial or ethnic information, symptoms of anxiety and depression in patients, and clinicians’ attitudes toward end-of-life care, which may introduce confounding factors. The lack of detailed socio-demographic data limits our ability to fully interpret differences between patients who received a serious illness conversation and those who did not, as these differences may reflect pre-existing patient or clinician characteristics rather than the effects of the conversations themselves. We were, however, able to adjust for age, sex, and subtype of hematological malignancy, enhancing the robustness of our analysis. As a single-center study, the results may have limited generalizability to other settings where the culture regarding engagement in serious illness conversations and end-of-life care may differ. Finally, no fidelity assessment was performed to document the quality of the conversations, reducing the ability to attribute associations to specific components of the conversations. Despite this, the implementation had robust management support, enabling comprehensive training for, and participation of all clinicians at the Department of Hematology. We have previously documented in a qualitative study that physicians and nurses experienced that interprofessional delivery strengthened their collaboration [21]. Additionally, the implementation of system changes, including allocated time for conversations and standardized documentation templates, further reinforced the integration and implementation of the conversations. Moreover, our inclusion of various hematological subtypes provides a more comprehensive understanding of the potential impact of serious illness conversations across a broad spectrum of hematological patients. Finally, our study is among the few to examine the associations between implementing serious illness conversations in real-world settings and end-of-life outcomes among patients with diverse hematological malignancies.

## Conclusion

Serious illness conversations are associated with a reduction in end-of-life anticancer treatment and an increase in referrals to specialized palliative care.

Building on these hypothesis-generating results, we have initiated a randomized controlled trial to validate and further explore these findings.

## Data Availability

No datasets were generated or analysed during the current study.
